# Species A rotavirus NSP3 acquires its translation inhibitory function prior to stable dimer formation

**DOI:** 10.1371/journal.pone.0181871

**Published:** 2017-07-24

**Authors:** Hugo I. Contreras-Treviño, Edgar Reyna-Rosas, Renato León-Rodríguez, Blanca H. Ruiz-Ordaz, Tzvetanka D. Dinkova, Ana M. Cevallos, Luis Padilla-Noriega

**Affiliations:** 1 Programa de Maestría y Doctorado en Ciencias Bioquímicas, Universidad Nacional Autónoma de México, Mexico City, Mexico; 2 Departamento de Microbiología y Parasitología, Facultad de Medicina, Universidad Nacional Autónoma de México, Mexico City, Mexico; 3 Departamento de Biología Molecular y Biotecnología, Instituto de Investigaciones Biomédicas, Universidad Nacional Autónoma de México, Mexico City, Mexico; 4 Departamento de Bioquímica, Facultad de Química, Universidad Nacional Autónoma de México, Mexico City, Mexico; Consejo Superior de Investigaciones Cientificas, SPAIN

## Abstract

Species A rotavirus non-structural protein 3 (NSP3) is a translational regulator that inhibits or, under some conditions, enhances host cell translation. NSP3 binds to the translation initiation factor eIF4G1 and evicts poly-(A) binding protein (PABP) from eIF4G1, thus inhibiting translation of polyadenylated mRNAs, presumably by disrupting the effect of PABP bound to their 3’-ends. NSP3 has a long coiled-coil region involved in dimerization that includes a chaperone Hsp90-binding domain (HS90BD). We aimed to study the role in NSP3 dimerization of a segment of the coiled-coil region adjoining the HS90BD. We used a vaccinia virus system to express NSP3 with point mutations in conserved amino acids in the coiled-coil region and determined the effects of these mutations on translation by metabolic labeling of proteins as well as on accumulation of stable NSP3 dimers by non-dissociating Western blot, a method that separates stable NSP3 dimers from the monomer/dimerization intermediate forms of the protein. Four of five mutations reduced the total yield of NSP3 and the formation of stable dimers (W170A, K171E, R173E and R187E:K191E), whereas one mutation had the opposite effects (Y192A). Treatment with the proteasome inhibitor MG132 revealed that stable NSP3 dimers and monomers/dimerization intermediates are susceptible to proteasome degradation. Surprisingly, mutants severely impaired in the formation of stable dimers were still able to inhibit host cell translation, suggesting that NSP3 dimerization intermediates are functional. Our results demonstrate that rotavirus NSP3 acquires its function prior to stable dimer formation and remain as a proteasome target throughout dimerization.

## Introduction

Species A rotavirus (RVA) is a double-stranded RNA virus of the *Reoviridae* family. RVA is a major cause of gastroenteritis in infants and in the young of several animal species. The genome of this virus has 11 segments that code for 12 proteins: six structural that form the triple-layered capsid (VP1-VP4, VP6 and VP7) and six non-structural (NSP1-NSP6) that participate in viral replication and host cell infection [1, 2].

RVA is useful as a model to study the relevance of the poly-(A) tail in translation of cellular mRNAs because rotaviral mRNAs lack this feature. Upon internalization into the host cell cytoplasm, the outer layer of the virion is lost, and the double-layered particle functions as a transcription machine that simultaneously produces the 11 viral mRNAs capped at the 5’-end and with the 3’-end sequence–UGACC. The viral mRNAs are either translated in the cytoplasm or used as templates to synthesize the dsRNA genome in cytoplasmic inclusion bodies known as viroplasms [3].

NSP3 of RVA is a 34-kDa protein with two major separate domains: an RNA-binding domain at the amino-terminus (RBD, amino acids 4–149) that binds to the 3’-terminal sequence–UGACC of viral mRNAs; and a translation initiation factor eIF4G1-binding domain (GBD, amino acids 206–313) localized at the carboxy-terminus. The NSP3 RBD and GBD are connected via a segment of the protein that is sufficient for interaction in a yeast two-hybrid system (HID [two-hybrid interaction domain], amino acids 150–206) [4]. The structures of the RBD and GBD have been separately determined by X-ray crystallography, and interestingly, the RBD is an asymmetric homodimer with a single RNA-binding tunnel, whereas the GBD is a symmetric homodimer with two hydrophobic eIF4G1 binding sites [5, 6]. Based on the ability of NSP3 to bind RVA mRNAs *in vitro* and to evict PABP from eIF4G *in vitro* and *in vivo*, Poncet and colleagues proposed two mechanisms by which NSP3 inhibits the translation of cellular mRNAs and also enhances the translation of rotaviral mRNAs. First, binding of NSP3 to eIF4G1 participating in translation initiation would inhibit polyadenylated mRNA translation by disruption of the 5’ to 3’ mRNA interaction that occurs via eIF4G1 bound to eIF4E and PABP [7]. Second, NSP3 simultaneously binding to the 3’ end of RVA mRNA and eIF4G1 would enhance translation of rotavirus mRNAs, thus functioning as an analogue of PABP [8]. Recently, a third function of NSP3 as a surrogate of PABP was proposed based on the finding that NSP3 enhances translation of transfected polyadenilated and nonpolyadenilated mRNAs and favor eIF4E-eIF4G interaction [9].

The effect of RVA NSP3 on host cell translation has been studied with different methods that are difficult to compare. In an *in vitro* assay with rabbit reticulocytes programmed with endogenous mRNAs from HeLa cell extracts and complemented with purified recombinant NSP3_163-313_, the truncated protein, containing the GBD, inhibited cellular translation [10]. Moreover, in an *in vivo* expression system of mammalian cells infected with a recombinant vaccinia virus for expression of NSP3, the full-length and a truncated protein, containing the GBD, inhibited host cell translation [11]. By contrast, using an *in vivo* approach based on transient cytoplasmic NSP3 expression programmed by electroporation with synthetic polyadenylated and nonpolyadenilated mRNAs, NSP3 enhanced translation of the transfected mRNAs regardless of their 3’ end [9]. This *in vivo* approach based on electroporation of synthetic mRNAs was also used to demonstrate that NSP3 enhances translation of RVA-like mRNAs, *i*.*e*., capped and with the 3’-end sequence–UGACC [12]. It is unclear why NSP3 inhibits host cell translation under some conditions but enhances host cell translation under other conditions.

Interestingly, expression of recombinant NSP3 redirects cytoplasmic PABP to the nucleus, a phenotype that has been proposed to contribute to host cell translation inhibition [13]. One study showed that interaction of NSP3 with eIF4G1 and the cellular protein RoXaN are both required for nuclear localization of PABP [14]. RoXaN is an AU-rich element (ARE)-binding protein and is thus expected to bind AREs at the 3’ untranslated region of many short lived mRNAs that serve as regulatory elements for mRNA degradation and translation [15]. Expression of a deletion NSP3 mutant, unable to bind eIF4G1 due to the lack of part of the carboxy-terminus GBD, restored cytoplasmic localization of PABP. In addition, expression of two NSP3 point mutations in the RoXaN-binding domain (RoBD, amino acids 163–237), that affected the ability to bind RoXaN as determined by co-immunoprecipitation, led to reduced nuclear localization of PABP compared to the wild type protein [14].

NSP3 has a long coiled-coil region (amino acids 159–245) predicted by the Lupas algorithm [4, 16] that is relevant for the dimerization of the protein. A segment of this region is sufficient to multimerize in a yeast two-hybrid system (HID, amino acids 150–205) [4]. Another segment is needed for binding to a chaperone, the Hsp90 binding domain (HS90BD, amino acids 225–258) [17]. Overlapping with the HS90BD, the coiled-coil participates in the dimer interface of the GBD, as determined by X-ray crystallography (amino acids 205–245) [6]. Coiled-coils are structural motifs common in dimers in which two alpha helices wrap around each other in a left-handed helix to form a supercoil. Coiled-coils have a periodicity of seven (heptad repeat), usually denoted (a-b-c-d-e-f-g)_n_ in one helix and (a’-b’-c’-d’-e’-f’-g’)_n_ in the other, where “a” and “d” are typically nonpolar residues found at the core of the interface of the two helices and “e” and “g” are solvent-exposed polar residues that provide specificity between the two helices through electrostatic interactions. The remaining positions, “b”, “c” and “f”, lie away from the coiled-coil interface, and tend to be occupied by polar residues [16].

Chawla-Sarkar and colleagues demonstrated that NSP3-Hsp90 interaction is essential for NSP3 dimerization and for the acquisition of function. As determined by *in vitro* transcription-translation assays, the expression of NSP3 in the presence of the Hsp90 inhibitor 7-N,N-dimethylethylenediamine-geldanamycin (17DMAG) prevented dimer formation, as did expression of NSP3 lacking the HS90BD. In cells infected with RVA, treatment with 17DMAG impaired the nuclear localization of PABP [17]. Moreover, Chawla-Sarkar and colleagues identified intermediates in the process of dimerization of NSP3 assisted by Hsp90. *In vitro* translated NSP3 molecules interacting with Hsp90 were immunoprecipitated with anti-Hsp90. In spite of being dimerization intermediates, the NSP3 molecules interacting with Hsp90 were detected as monomers by non-dissociating electrophoresis [17]. Based on the demonstration that NSP3 dimerization intermediates migrate as monomers by non-dissociating Western blot (WB-ND), we refer to the apparently monomeric forms of the protein detected by this method as monomers/dimerization intermediates. Further incubation of immunoprecipitated NSP3-Hsp90 complexes with fresh translation mixture showed that NSP3 molecules were converted to stable dimers free of Hsp90, as determined by WB-ND. Interestingly, in the presence of 17DMAG, *in* vitro translated NSP3 monomers were readily degraded, suggesting that *bona fide* NSP3 monomers are short lived [17].

The recent finding of polyadenylated mRNA translation enhancement by RVA NSP3 challenges our notion about the role of this protein, that has long been considered an inhibitor of host cell translation [9, 12]. We hypothesized that the process of dimerization is a key in understanding the mechanism by which NSP3 affects host cell translation. In this study, we used a vaccinia virus system to express wtNSP3 and NSP3 carrying point mutations in a segment of the coiled-coil region and evaluated their dimerization status, effects on cellular translation, and stability to the proteasome. Our results demonstrate that NSP3 acquires its function prior to stable dimer formation.

## Materials and methods

### Reagents and antibodies

The inductor isopropyl β-D-1-thiogalactopyranoside (IPTG, cat. 70571) was obtained from Research Organics (Cleveland, OH). Proteasome inhibitor MG132 (cat. C2211) and cycloheximide (cat. C7698) were obtained from Sigma-Aldrich (St. Louis, MO). The horseradish peroxidase conjugated chicken anti-rat IgG (cat. SC2956) was obtained from Santa Cruz Biotechnology (Dallas, TX). The hyperimmune anti-NSP3 rat serum was produced in our laboratory.

### Cells, viruses, and plasmids

*Cercopithecus aethiops* CV-1 and BSC-1 cell lines were obtained from R. Rosales (Universidad Nacional Autónoma de México, Mexico City, Mexico). The human HeLa cell line was obtained from A. González-Noriega (Universidad Nacional Autónoma de México, Mexico City, Mexico). The vaccinia virus vT7lacOI and plasmid p.VOTE.1 were obtained from B. Moss (National Institutes of Health, Bethesda, MD). Vaccinia virus vRRV7 containing RRV gene 7 for expressing wild type NSP3 was previously produced in our laboratory [11]. CV-1, BSC-1 and HeLa cells were cultured in Eagle's minimum essential medium (MEM) supplemented with 7% of fetal bovine serum (Biowest, Mexico).

### Mutagenesis and recombinant vaccinia virus construction

Site-directed mutagenesis for expression in the VOTE (vaccinia virus/*lac* operon/T7 RNA polymerase/EMC IRES) mammalian cell expression system [18] was performed with the QuickChange Site-Directed Mutagenesis kit (Agilent Technologies, Santa Clara, CA) according to the manufacturer’s instructions. The plasmid p.VOTE-RRV7 [11] for the expression of the NSP3 gene of the simian RVA strain RRV (GenBank AY065842) was mutagenized using five oligonucleotide pairs to produce the plasmids p.VOTE-RRV7-W170A (ROXF7170, GGAAGTAGATACAATTGATGCCAAATCAAGATATGAACAG; ROXR7170, CTGTTCATATCTTTGATTTGGCATCAATTGTATCTACTTCC), p.VOTE-RRV7-K171E (ROXF7171, GGAAGTAGATACAATTGATTGGGAGTCAAGATATGAACAGTTAG; ROXR7171, CTAACTGTTCATATCTTGACTCCCAATCAATTGTATCTACTTCC), p.VOTE-RRV7-R173E (ROXF7173, GATACAATTGATTGGAAATCAGAGTATGAACAGTTAGAAAAGAG; ROXR7173, CTCTTTTCTAACTGTTCATACTCTGATTTCCAATCAATTGTATC), p.VOTE-RRV7-R187E:K191E (ROXF7187, GAGTCACTGAAACATGAGGTTAATGAGGAGTATAATCATTGGG; ROXR7187, CCCAATGATTATACTCCTCATTAACCTCATGTTTCAGTGACTC), and p.VOTE-RRV7-Y192A (ROXF7192, CATCGGGTTAATGAGAAGGCCAATCATTGGGTTCTTAAAGC; ROXR7192, GCTTTAAGAACCCAATGATTGGCCTTCTCATTAACCCGATG). The NSP3 gene inserts in the corresponding plasmids were sequenced in both directions. The plasmids were then used to generate five recombinant vaccinia viruses by homologous recombination in CV-1 cells infected with the parental vaccinia virus vT7lacOI, as previously described [19]: vNSP3_W170A_, vNSP3_K171E_, vNSP3_R173E_, vNSP3_R187E:K191E_, and vNSP3_Y192A_. In the VOTE system, the *lacI* gene is transcribed from a vaccinia early/late promoter, resulting in constitutive expression of the *E*. *coli lac* repressor, which binds to two *E*. *coli lacO* operators, one adjacent to a vaccinia virus late promoter to prevent transcription from the bacteriophage T7 RNA polymerase gene and the other next to a T7 promoter to prevent transcription of the NSP3 gene. Upon induction with IPTG, the repressor is inactivated, allowing expression of the T7 RNA polymerase, which then transcribes the NSP3 gene. Finally, the translation of the NSP3 mRNA is enhanced by an encephalomyocarditis virus internal ribosome entry site (IRES). Vaccinia virus stocks were produced in HeLa cells and titrated for plaque forming units in BSC-1 cells, as previously described [19].

### Metabolic labeling of proteins in vaccinia virus-infected cells

We performed metabolic labeling with [^35^S]-methionine plus [^35^S]-cysteine in cells infected with vaccinia viruses as previously described [19] with some modifications. BSC-1 cells in MEM containing 7% FBS were plated in 6-well plates (5 x 10^5^/well) and incubated 12–24 h at 37°C. The cells were infected with the recombinant vaccinia viruses with a multiplicity of infection (MOI) of five. At two hours post-infection (hpi), the inducer IPTG (0.4 mM, unless otherwise specified) and the proteasome inhibitor MG132 (0.5 μM) or its diluent dimethyl sulfoxide (DMSO) alone were added as indicated and maintained during expression. At 17.5 hpi, the cells were starved for 30 min at 37°C with methionine- and cysteine-free DMEM (Invitrogen, Carlsbad, CA). Labeling was performed by adding 20 μCi/well of [^35^S]-methionine and [^35^S]-cysteine mixture (EasyTag^™^ EXPRESS35S Protein Labeling Mix, Perkin Elmer, Norwalk, CT) in 500 μL of methionine- and cysteine-free DMEM. The cells were incubated for 1 h at 37°C and harvested by washing twice with cold PBS and scraping in 1 mL of cold PBS. The cells were divided in two 0.5-mL aliquots and centrifuged at 13,200 g for 5 min. The cell pellets were suspended in 20 μL of lysis buffer (50 mM Tris-HCl pH 7.5, 5 mM MgCl_2_, 0.4 U/mL DNase I, 0.01 mg/mL RNase A and Complete^®^ protease inhibitor [Roche]), and incubated for 10 min at room temperature. The cell lysates were mixed with 20 μL of 2x Laemmli sample buffer (62.5 mM Tris-HCl pH 6.8, 20% glycerol, 4% SDS and 2% β-mercaptoethanol) and heated in boiling water bath for five minutes. Labeled proteins were separated in 12% SDS-PAGE and analyzed by autoradiography and densitometry. Molecular weight assessment was based on predominant late vaccinia virus proteins detected in cells infected with vT7lacOI [20]. Analysis to determine the effect of NSP3 on host cell translation was done by quantitating the densitometry profile (excluding the NSP3 band) of three independent experiments performed on different days. Identical aliquots were processed as loading controls by running SDS-PAGE and analyzing the protein content by silver staining, using a variant protocol optimized for protein quantification [21].

### Western blot (WB) and non-dissociating Western blot (WB-ND)

BSC-1 cells were infected with vaccinia viruses and treated or not with MG132 as described above. At 18 hpi, the cells were harvested by washing twice with cold PBS and scraping in 1 mL of cold PBS. The cells were divided in four 0.25-mL aliquots and centrifuged at 13,200 g for 5 min. The cell pellets were suspended in 20 μL of lysis buffer and incubated for 10 min at room temperature. For dissociating conditions (WB), the cell lysates were mixed with 20 μL of 2x Laemmli sample buffer and heated in boiling water bath for five minutes. For non-dissociating conditions (WB-ND), the cell lysates were mixed with 20 μL of 2x non-dissociating sample buffer (62.5 mM Tris-HCl pH 6.8, 0.8% SDS, 20% glycerol) and incubated for 30 minutes on ice. The proteins were separated in 12% SDS-PAGE (run in a cold room for WB-ND) and transferred to polyvinylidene fluoride membranes (BioRad, Hercules, CA, cat. 162–0177). One lane of prestained protein markers (New England Biolabs, Ipswich, MA, cat. P7708S) was used to estimate the size of proteins. The membranes were blocked with 5% non-fat dry milk in Tris-buffered saline (TBS) at room temperature for one hour. The membranes were incubated overnight at 4°C with anti-NSP3 (1:20,000) in 1% non-fat dry milk in T-TBS (0.05% Tween-20 in TBS), followed by incubation with horseradish peroxidase-conjugated anti-rat IgG (1: 20,000) in 1% non-fat dry milk in T-TBS for two hours at room temperature. Finally, the blots were developed with SuperSignal West Femto chemiluminescence substrate (Pierce, Rockford, IL) according to the manufacturer’s instructions. A detailed protocol for determining the half-life of the dimeric form of NSP3 by cycloheximide blocking and WB-ND is available at dx.doi.org/10.17504/protocols.io.iiwccfe. Densitometry analysis of three independent experiments performed on different days was done by determining the pixels of the low molecular weight band consisting of NSP3 monomers/dimerization intermediates and the high molecular weight band consisting of NSP3 stable dimers, excluding the smear of putative poly-ubiquitinated dimers that migrates above the dimer band. Identical aliquots were processed as loading controls by running SDS-PAGE and analyzing the protein content by silver staining using a variant protocol optimized for protein quantification [21].

The half-lives of monomeric and stable dimeric NSP3 were determined by incubation in the presence of a protein synthesis inhibitor and quantification of protein decay by WB-ND. This method differs from half-life determination by pulse-chase assays in that the whole accumulated NSP3 contributes to the signal detected by WB rather than the recently synthetized protein labelled with radioactive amino acids during the pulse. BSC-1 cells were infected with vNSP3, adding IPTG and MG132 or its diluent as indicated above. At 14 hpi, the medium was removed, the cells were washed twice with MEM containing 2.5% SFB, and cycloheximide (100 μg/mL) was added in MEM containing 2.5% SFB. The cells were harvested immediately as described above (time 0) or incubated for 30, 60, 120 and 240 minutes and then harvested. The cells were processed by WB-ND with anti-NSP3 serum.

### Nucleotide sequence analysis

Nucleotide sequences of the NSP3 gene from several RVA strains were aligned with Clustal Omega [22].

## Results

### Production of RVA NSP3 with point mutations in the coiled-coil region for expression in the vaccinia VOTE system

**Fig 1:**
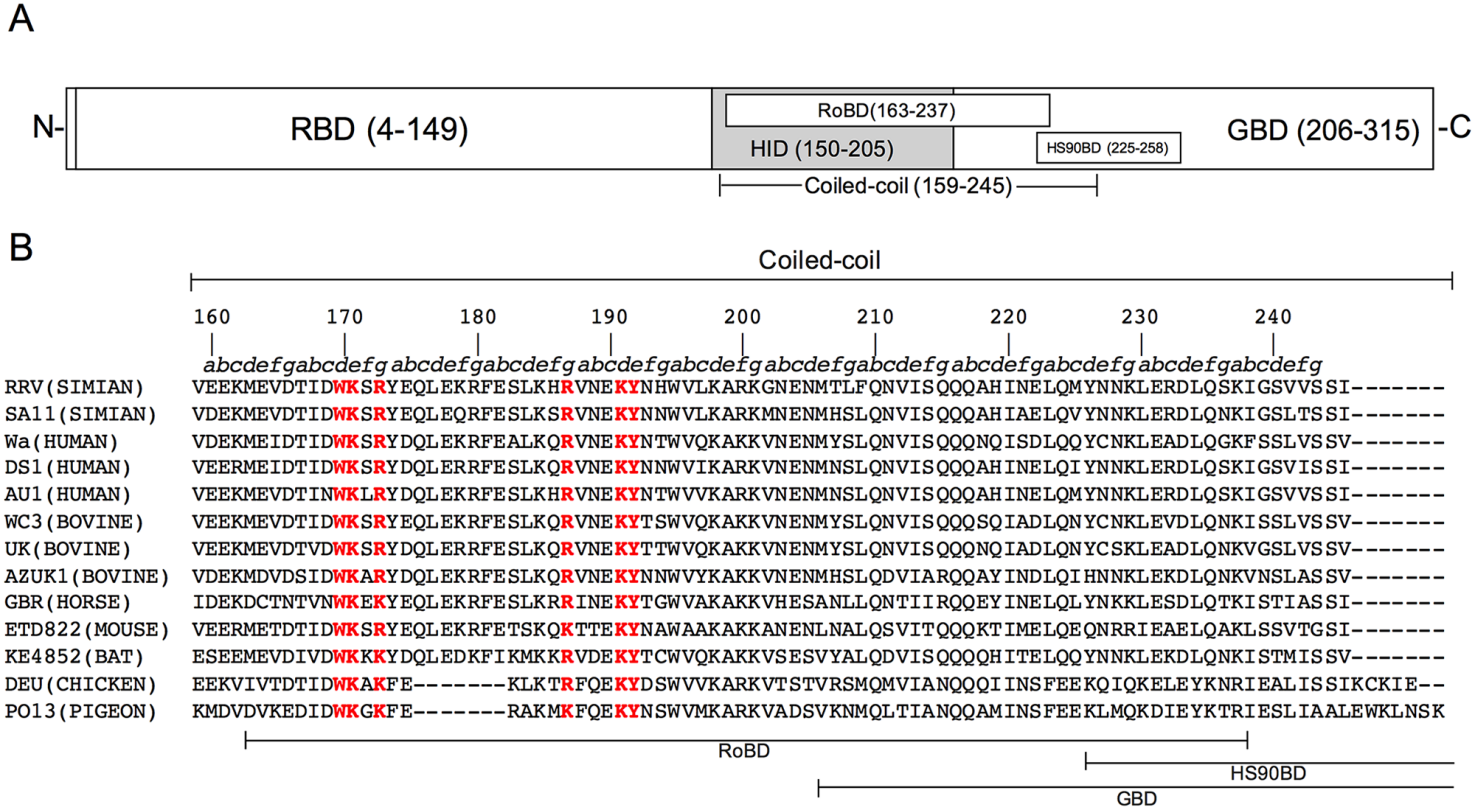
Mutations produced in the coiled-coil region of the RRV NSP3 gene and comparison with the sequence of other RVA strains. (A) Schematic representation of NSP3 domains: RBD, RNA-binding; RoBD, RoXaN-binding; HID, two-hybrid interaction; HS90BD, Hsp90-binding; GBD, eIF4G1-binding. The lower line indicates the coiled-coil region, and the numbers refer to the NSP3 amino acid sequence. (B) Alignment of the amino acid sequences of the coiled-coil region of the NSP3 gene of RRV and other RVA strains that infect diverse animal species. The heptad repeat pattern (a-g)_n_ is shown above, where “a” and “d” are hydrophobic and “e” and “g” are charged residues. The remaining positions, “b”, “c” and “f”, tend to be occupied by polar residues. Mutagenized residues are highlighted in red. The lower lines indicate the segments of the coiled-coil region that overlap with NSP3 domains.

To begin to study the role of the coiled-coil region of RVA NSP3 in dimerization, we performed site-directed mutagenesis of conserved amino acids in a segment of the coiled-coil region adjoining the HS90BD. We used the vaccinia virus VOTE system to express the NSP3 gene of the simian RVA strain RRV [11, 18]. The VOTE system depends on infection of mammalian cells by recombinant vaccinia viruses carrying the gene of interest, allowing high levels of IPTG-inducible expression. Mutagenesis was first performed using the plasmid p.VOTE-RRV7 previously constructed for the expression of full-length NSP3 of RRV (amino acids 4–313) [11]. In this construct, the expressed protein lacks amino acids 1–3 due to initiation at the second initiation codon because the first in-frame initiation codon is in a poor context. After performing mutagenesis in the plasmids, five recombinant vaccinia viruses for expression of the mutant proteins (vNSP3_W170A_, vNSP3_K171E_, vNSP3_R173E_, vNSP3_R187E:K191E_, and vNSP3_Y192A_) were produced in CV-1 cells infected with the parental vaccinia virus vT7lacOI, as described in materials and methods. Fig 1 shows the mutations produced in the context of the amino acid sequence of the coiled-coil segment of NSP3 from RRV, which is compared with the corresponding sequences of other RVA strains from different species. One mutation was produced in a “d” residue of the heptad repeat (W170A). Based on its position, it was expected to be at the core of the interface between the two helices of the dimer. Three mutations were produced at “e” (K171E and Y192A) or “g” (R173E) residues and were accordingly expected to affect the specificity between the two helices of the dimer. The double mutation (R187E:K191E) at “g” and “d” residues was expected to affect simultaneously the interface of the dimer and the specificity of the interaction. The double mutant R187E:K191E combined two mutations that have been previously assessed individually in the NSP3 gene of the bovine RVA strain RF, where they were shown to affect the interaction with RoXaN by co-immunoprecipitation [14]. In this initial mutagenesis study, we targeted a few amino acids in the lengthy coiled-coil of NSP3 expected to have a range of effects on dimerization, including a double mutation known to affect the binding to a cellular protein. Our hypothesis was that mutations in “d” residues would interrupt the repeated hydrophobic interactions at the helical interface, thus having a stronger effect than mutations in “e” or “g” residues that affect interhelical ionic interactions and contribute marginally to the stability of the dimer. None of the mutations affected the HS90BD.

### Effect of mutations in the coiled-coil region of RRV NSP3 on host cell translation

We determined the effect of the RRV NSP3 mutations on mammalian host cell translation using the vaccinia virus VOTE system [18]. Cells were infected with the recombinant vaccinia virus followed at 18 hpi by pulse labeling with [^35^S]-labeled amino acids, SDS-PAGE of the harvested cells, autoradiography and densitometry analysis. This method was used to determine the combined effect of NSP3 on cellular and vaccinia virus mRNAs that are both capped and polyadenylated. We have previously used the VOTE system to demonstrate that NSP3 inhibits host cell translation [11]. However, in the present study, we avoided hypertonic conditions during metabolic labeling, reduced the concentration of the inducer, and shortened the time of infection. Despite lowering the concentration of the inducer and shortening the time of infection, we still achieved maximum inhibition of host cell translation (S1 and S2 Figs). Hypertonic condition with higher NaCl concentration in the culture medium favors the expression of IRES-driven genes [23] and has a synergic effect with the inhibitory function of NSP3 on host cell translation, thus explaining the difference in maximum host cell translation inhibition obtained in this study (see below and S2 Fig) as compared to our previous studies [11].

**Fig 2:**
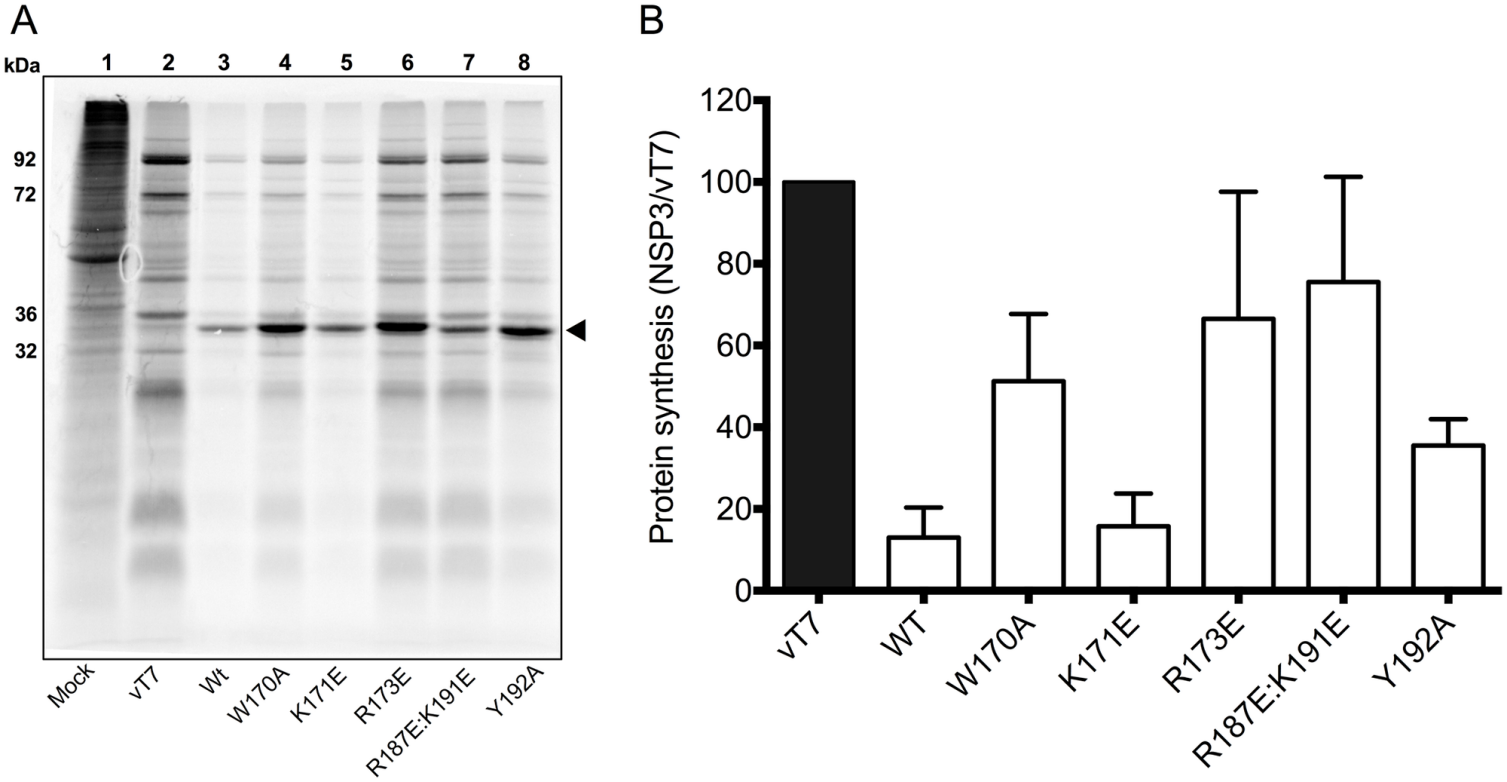
Effect of point mutations in the coiled-coil region of RRV NSP3 on the inhibitory function of NSP3 in host cell translation. BSC-1 cells were mock-infected, infected with the parental virus vT7lacOI, or with viruses for the expression of wild type NSP3 or its mutants with a MOI of five. At two hpi the inducer IPTG was added (0.4 mM). The infected cells were pulse labeled with [^35^S]-methionine plus [^35^S]-cysteine prior to harvesting at 18 hpi. The cells were then analyzed by SDS-PAGE and autoradiography (A). The molecular weights of four predominant vaccinia virus proteins detected in lane 2 are indicated to the left. The solid triangle indicates the position of the NSP3 bands (34 kDa). Based on densitometry analysis of three independent experiments, the graph bars (B) indicate the percentage of protein synthesis in cells expressing NSP3 or its mutants compared with control cells that do not express NSP3 (vT7). Bars indicate standard deviation.

*Cercopithecus aethiops* BSC-1 cells were infected with the parental control virus vT7lacOI lacking the NSP3 gene or with viruses for expression of wtNSP3 or its mutants, and their effects on host cell translation were determined in the presence of the inducer IPTG (0.4 mM). Cells infected with the parental virus vT7lacOI exhibited the characteristic pattern of cellular and late vaccinia virus proteins, which differs from the cellular proteins synthesized in mock-infected cells (Fig 2A, lanes 1 and 2). Cells infected with vNSP3 for the expression of wtNSP3 or with any of the five viruses for the expression of NSP3 mutants showed a prominent 34-kDa band that corresponds to the molecular weight of NSP3 (Fig 2A, lanes 3–8). In a previous study, we demonstrated that this 34-kDa band corresponds to NSP3 by immunoprecipitation with hyperimmune anti-NSP3 serum [11]. Compared with BSC-1 cells infected with the parental vT7lacOI virus, infection with the viruses for the expression of wtNSP3 or its mutants had different effects on host cell translation. As determined by densitometry analysis of three independent experiments (Fig 2B), expression of wtNSP3 inhibited 87% of host cell translation. The NSP3 harboring mutation K171E inhibited translation to a similar extent as wtNSP3 (84%). The other four mutations inhibited host cell translation moderately (64% Y192A and 49% W170A) or poorly (33% R173E and 24% R187E:K191E). Similar results were obtained using increasing doses of the inducer IPTG (S1 Fig). As observed in Fig 2A, the amount of wtNSP3 or its mutants synthesized *de novo* during the [^35^S]-labeled amino acids-pulse (1 h) was widely variable. However, the observed differences were not the same as those of overall protein accumulated during the 18 h elapsed from the time of infection (see below).

### Accumulation of wtNSP3 and its mutants and susceptibility to a proteasome inhibitor

**Fig 3:**
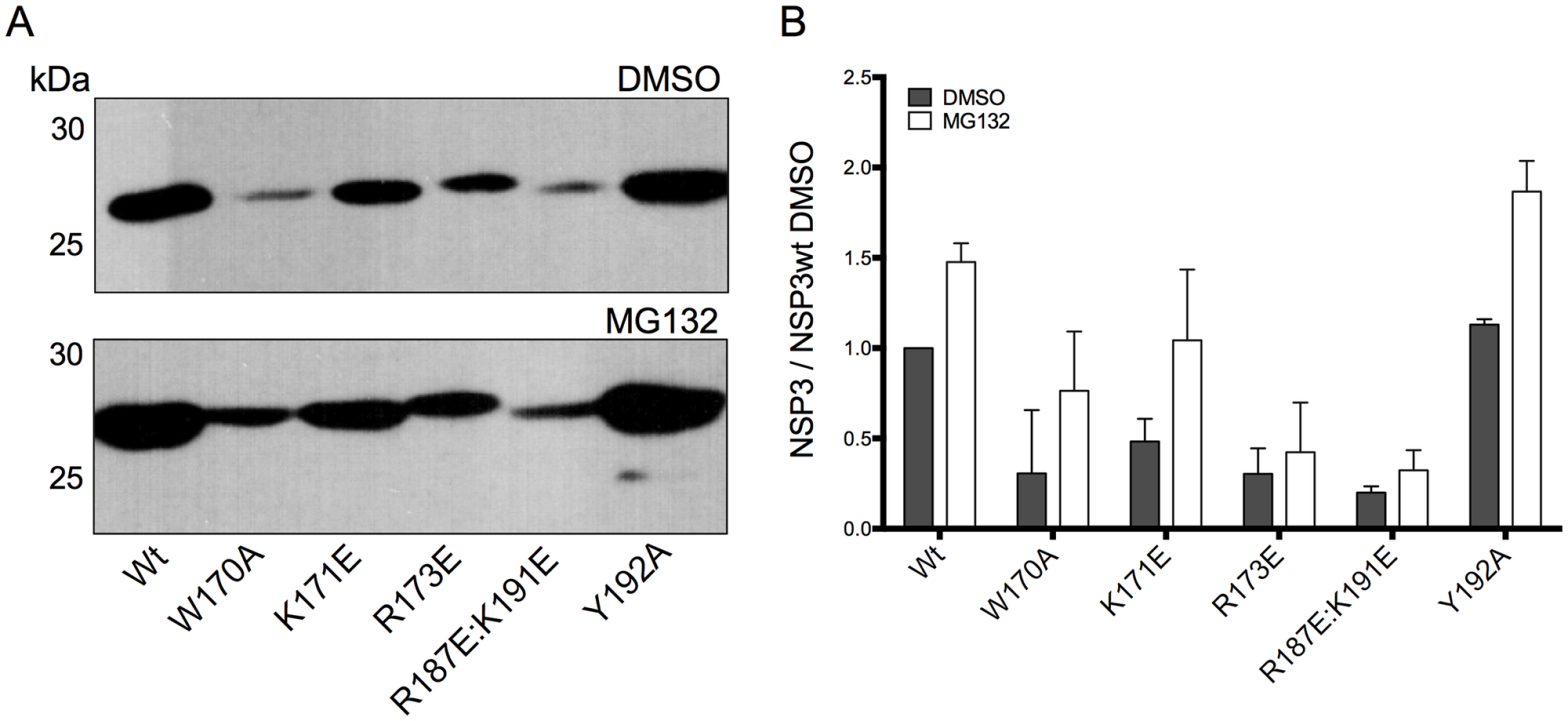
Effect of point mutations in the coiled-coil region of RRV NSP3 on its accumulation and susceptibility to the proteasome. Identical numbers of BSC-1 cells were infected with the parental virus vT7lacOI or with viruses for the expression of wild type NSP3 or its mutants. The cells were incubated with a proteasome inhibitor (MG132) or with its diluent (DMSO). The infected cells were harvested at 18 hpi and analyzed by WB with anti-NSP3 serum (A). Based on densitometry analysis of three independent experiments, the graph bars (B) indicate the accumulation of NSP3 in the presence of MG132 (white bars) or its diluent DMSO (gray bars) compared with the accumulation of the wild type protein in the absence of MG132 (Wt). Bars indicate standard deviation.

We next sought to quantify the accumulation of wtNSP3 and its mutants at the post-infection time point used to determine their effects on host cell translation. In addition, we wanted to determine the effect of the proteasome on the accumulation of these proteins. We therefore infected BSC-1 cells with viruses to express wtNSP3 and its mutants, followed by the addition of the proteasome inhibitor MG132 or its diluent DMSO, and after 16 h of incubation with the drug, the cells were harvested and analyzed by Western blot (WB) with anti-NSP3 serum (Fig 3A). As determined by densitometry analysis of three independent experiments, the amount of accumulated NSP3 varied widely among the mutants (Fig 3B). In the absence of proteasome inhibitor, four mutants accumulated lower NSP3 levels as compared to wtNSP3 (W170A, K171E, R173E and R187E:K191E), and one mutant accumulated a higher level (Y192A). The treatment with proteasome inhibitor MG132 increased the accumulated wtNSP3, and the same happened to all of the mutant proteins, and under these conditions, only the mutant Y192A accumulated a higher level of the mutant protein than wtNSP3, similar to the results obtained in the absence of MG132. These findings indicate that wtNSP3 and all of its mutants are susceptible to the proteasome to some degree. Moreover, susceptibility to the proteasome is likely a contributing factor that determines the low levels of accumulation of four mutants (W170A, K171E, R173E and R187E:K191E) and high level of accumulation of one mutant (Y192A).

### Accumulation of the stable dimer and monomer/dimerization intermediate forms of wtNSP3

**Fig 4:**
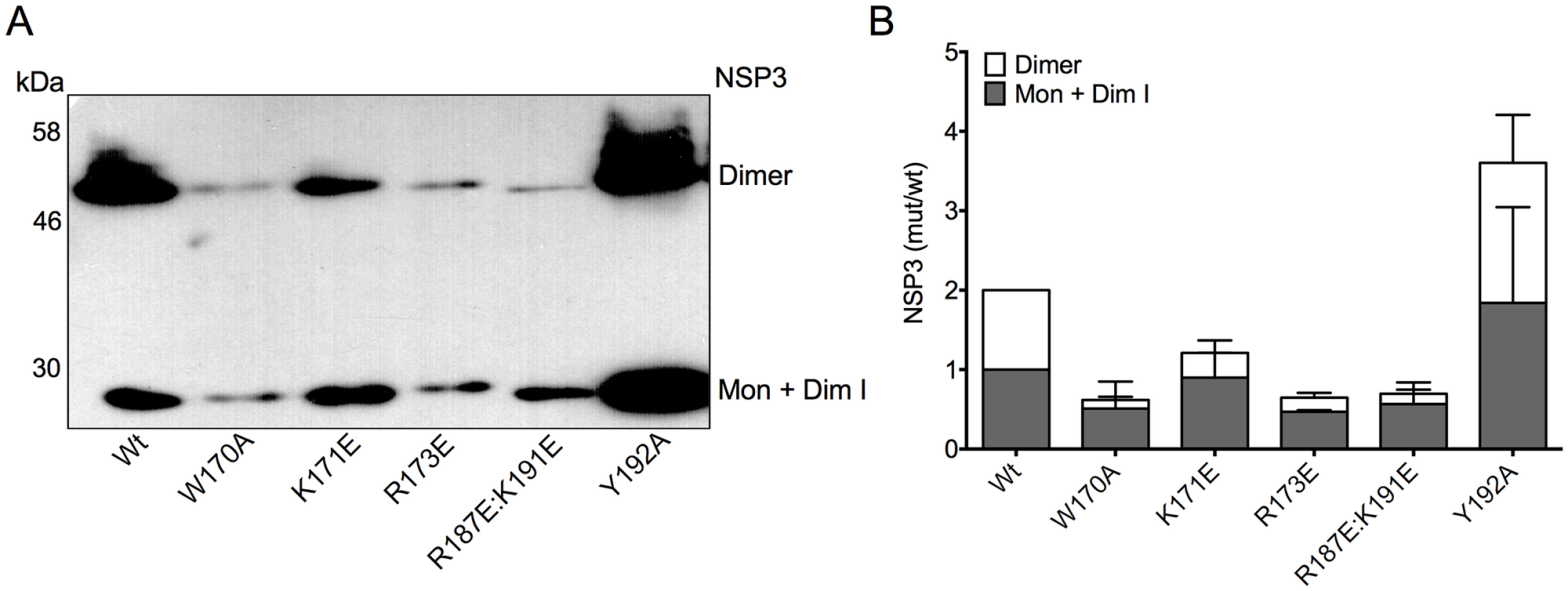
Effect of point mutations in the coiled-coil region of NSP3 on the accumulation of its stable dimeric and monomeric/dimerization intermediate forms. Identical numbers of BSC-1 cells were infected with vaccinia viruses for the expression of wild type NSP3 or its mutants. The infected cells were harvested at 18 hpi and analyzed by WB-ND with anti-NSP3 serum (A). The two bands correspond to monomeric/dimerization intermediate (34 kDa) and dimeric (68 kDa) forms of NSP3. Based on densitometry analysis of three independent experiments, the graph bars (B) indicate the accumulation of monomeric/dimerization intermediate and stable dimeric NSP3 compared with the accumulation of each one of these two forms of wtNSP3. Bar lines indicate standard deviation.

Because the mutations were designed to affect dimerization, we determined the accumulation of stable dimers of wtNSP3 and its mutants. For this purpose we used non-dissociating Western blot (WB-ND), a method previously described by Chawla-Sarkar and colleagues that uses low concentrations of SDS to separate stable NSP3 dimers from monomers/dimerization intermediates, these last two migrating as *bona fide* monomers that cannot be resolved [17]. BSC-1 cells were infected with viruses to express wtNSP3 and its mutants, and at 18 hpi the cells were harvested and analyzed by WB-ND. The accumulation of stable dimers and monomers/dimerization intermediates varied between wtNSP3 and its mutants (Fig 4A). Of interest, smears directly above the dimeric wtNSP3 and NSP3_Y192A_ bands were consistently observed but were excluded from the densitometry analysis. Based on three independent experiments, four of the NSP3 mutants accumulated lower levels of stable dimers as compared to wtNSP3 (W170A, K171E, R173E and R187E:K191E), and one mutant (Y192A) accumulated more (Fig 4B). The mutation that most severely affected the accumulation of stable dimers was NSP3_W170A_ (nine-fold lower than wtNSP3, and sixteen-fold lower than NSP3_Y192A_), and identical differences were obtained in further experiments that determined the accumulation of these proteins in the presence and absence of a proteasome inhibitor (see below). Consistent with the level of the stable dimeric form of the protein, the NSP3 mutants had variable levels of accumulated monomers/dimerization intermediates, with low levels for the mutations W170A, R173E and R187E:K191E, intermediate for the mutation K171E, and high for wtNSP3 and Y192A (Fig 4B). Notably, even though the mutant NSP3_Y192A_ accumulated a higher level of stable dimeric NSP3 than wtNSP3, inhibition of host cell translation was moderately affected as compared to the severe host cell translation inhibition caused by wtNSP3 (Fig 2). By contrast, the mutant NSP3_W170A_ had a severely reduced level of stable dimeric NSP3, but its function was moderately affected to a level that could not be distinguished from that of the mutant NSP3_Y192A_. Hence there was no correlation between the level of accumulation of stable dimeric NSP3 and its inhibitory function on host cell translation among the mutants. The finding that mutants severely impaired to form stable NSP3 dimers were still able to inhibit host cell translation indicates that in addition to the stable dimeric NSP3, the remaining NSP3 consisting of *bona fide* monomers and/or dimerization intermediates is somehow functional.

### Accumulation of the stable dimer and monomer/dimerization intermediate forms of wtNSP3 and its mutants in the presence of a proteasome inhibitor

**Fig 5:**
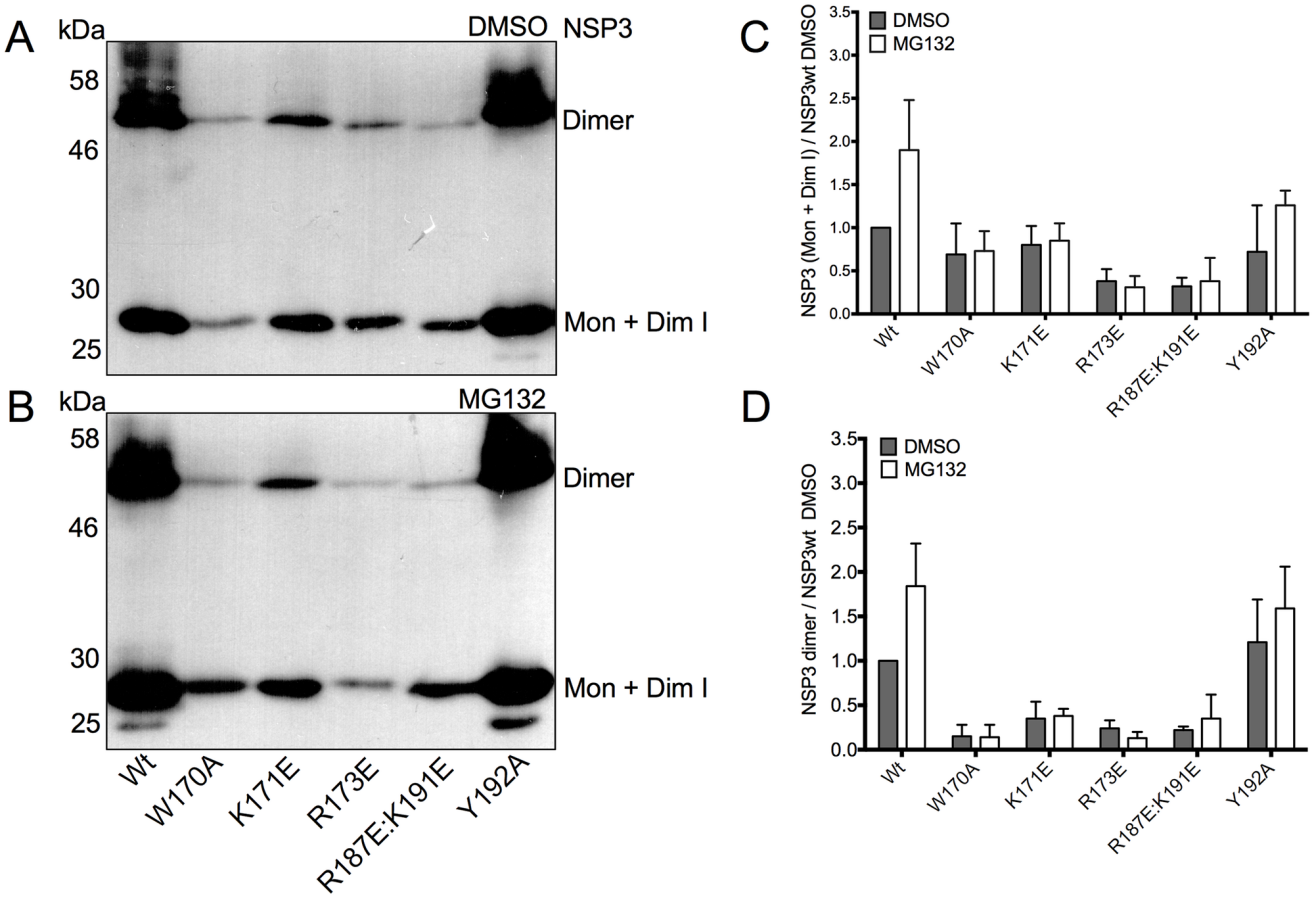
Effect of point mutations in the coiled-coil region of NSP3 on the accumulation of its stable dimeric and monomeric/dimerization intermediate forms in the presence and absence of MG132. Identical numbers of BSC-1 cells were infected with vaccinia viruses for the expression of wild type NSP3 or its mutants. The cells were incubated with a proteasome inhibitor (MG132) or its diluent (DMSO). The infected cells were harvested at 18 hpi and analyzed by WB-ND with anti-NSP3 serum (A and B). The two prominent bands correspond to monomeric/dimerization intermediate (34 kDa) and dimeric (68 kDa) forms of NSP3; the minor band may correspond to a putative degradation product (30 kDa). Based on densitometry analysis of three independent experiments, the graph bars compare the accumulation of monomers/dimerization intermediates (C) or stable NSP3 dimers (D) in the presence of MG132 (white bars) or its diluent (gray bars). Bars indicate standard deviation.

We next compared the accumulation of monomeric/dimerization intermediate and stable dimeric wtNSP3 and its mutants in the absence of MG132 with the accumulation of both forms of the protein in the presence of MG132. BSC-1 cells were infected with viruses to express wtNSP3 and its mutants, followed by the addition of the proteasome inhibitor MG132 or its diluent DMSO, and after 16 h of incubation with the drug, the cells were harvested and analyzed by WB-ND (Fig 5A and 5B). Based on three independent experiments, treatment with MG132 increased the level of the stable dimeric forms of wtNSP3 and NSP3_Y192A_ (Fig 5D). Coincidently, treatment with MG132 increased the level of the monomeric/dimerization intermediate forms of wtNSP3 and NSP3_Y192A_ (Fig 5C). By contrast, treatment with MG132 had no effect on the accumulation of the stable dimeric and monomeric/dimerization intermediate forms of four mutant proteins (W170A, K171E, R173E and R187E:K191E). Considering that all of the mutant NSP3 proteins were susceptible to the proteasome by WB (Fig 3), it is possible that WB-ND underestimates the effect of the proteasome on monomeric/dimerization intermediate and stable dimeric NSP3 because of an *in vitro* effect. WB-ND uses mild conditions to prepare cell extracts with a lower SDS concentration and maintains samples in the cold instead of boiling, and such conditions may not be able to fully prevent enzymatic activities that have an effect on the stability of NSP3, such as ubiquitination, Hsp90-assisted dimerization and proteasome degradation. Based on the observed differences in the apparent stability to *in vitro* conditions of four NSP3 mutants (W170A, K171E, R173E and R187E:K191E) that failed to accumulate higher levels of either dimeric and/or monomeric/dimerization intermediate forms of NSP3 in the presence of the proteasome inhibitor we hypothesize that the above mutations rendered proteins that are unstable *in vitro*.

Coincident with the high level of accumulation of the two forms of wtNSP3 and NSPY_192A_, a minor 30-kDa band increased its yield in the presence of MG132 (Fig 5A and 5B). This minor band is likely a degradation product of NSP3 that is recognized by the hyperimmune anti-NSP3 serum. In addition, high levels of accumulation of stable dimeric wtNSP3 and the mutant NSP3_Y192A_ were accompanied by a smear with some discernible bands that migrate just above the NSP3 dimers (Fig 5A and 5B). These smears were more prominent in cells treated with MG132 of either wtNSP3 of NSP3_Y192A_, thus suggesting that the smear consists of polyubiquitinated dimers. These putative polyubiquitinated NSP3 dimers were apparently absent in cells infected with four mutant viruses that exhibited reduced levels of accumulation of the monomeric/dimerization intermediate and stable dimeric forms. The above data are consistent with the notion that the low level of accumulation of the monomeric/dimerization intermediate and stable dimeric forms of the four NSP3 mutants (W170A, K171E, R173E and R187E:K191E) is due to higher susceptibility to the proteasome as compared with wtNSP3. By contrast, the high level of accumulation of NSP3_Y192A_ is likely due to lower susceptibility to the proteasome as compared to wtNSP3.

### Dimeric wtNSP3 is susceptible to proteasome degradation and has a shorter half-life than monomers and/or dimerization intermediates

**Fig 6:**
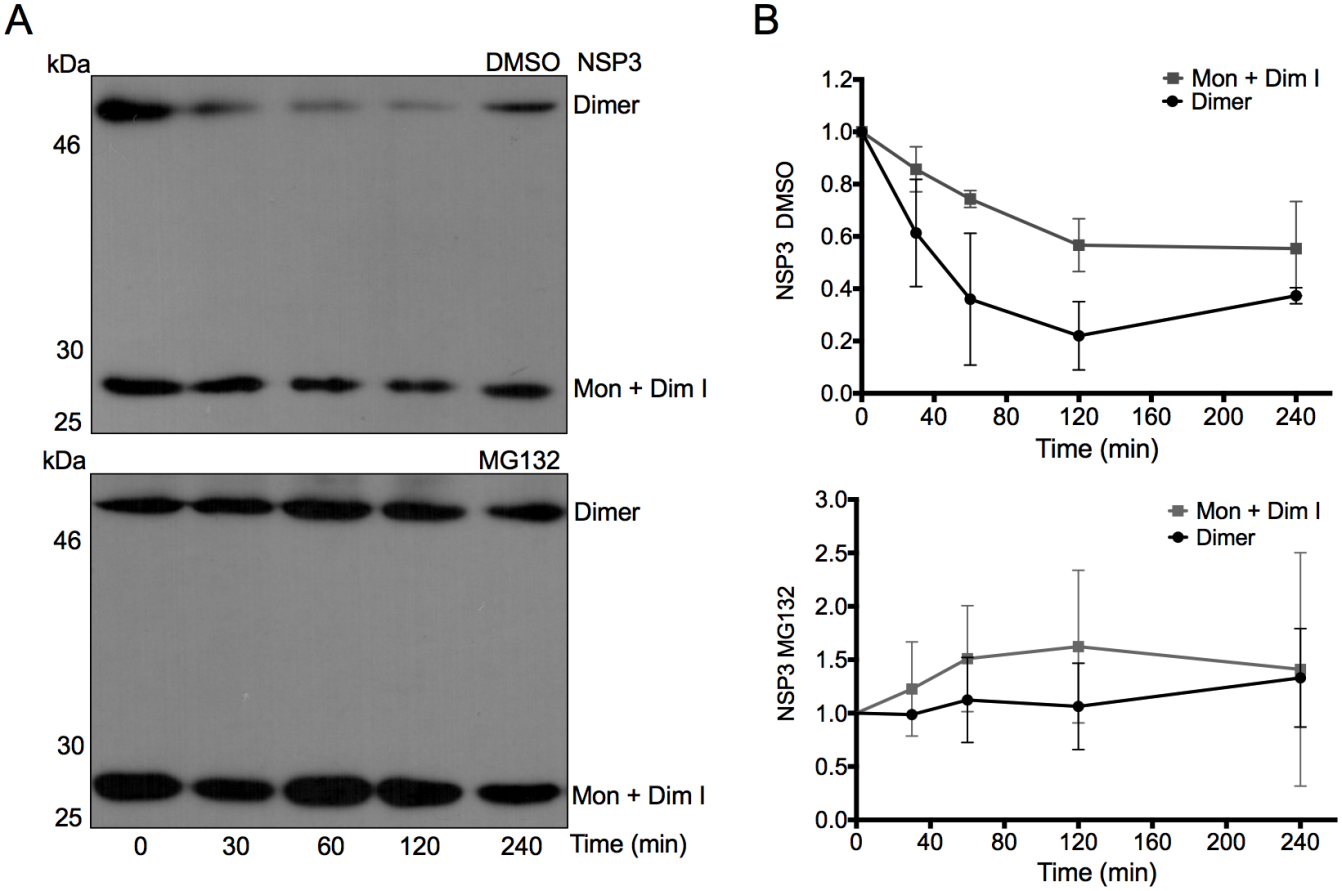
Determination of the half-lives of stable dimeric and monomeric/dimerization intermediate forms of wtNSP3 in the presence and absence of MG132. Identical numbers of BSC1 cells were infected with vNSP3 to express wtNSP3. At two hpi, the cells were treated with MG132 or its diluent DMSO, and at 14 hpi cycloheximide (CHX) was added to inhibit translation. The cells were harvested 0, 30, 60, 120 and 240 min after adding CHX. The harvested cells were analyzed by WB-ND with anti-NSP3 serum (A). The two bands correspond to the monomeric/dimerization intermediate (34 kDa) and dimeric (68 kDa) forms of NSP3. Based on densitometry analysis of three independent experiments, the graph lines (B) were used to determine the half-lives of the monomeric/dimerization intermediate (134 min) and stable dimeric (60 min) forms of the protein in the absence of MG132. Both forms of the protein were more stable in the presence of MG132. Bar lines indicate standard deviation.

The results in Fig 5 paradoxically failed to show directly an effect of the proteasome on the accumulation of the monomeric/dimerization intermediate and stable dimeric forms of four NSP3 mutants in spite of the fact that proteasome degradation is the major determinant of the level of accumulation of wtNSP3 [17]. We used a different approach to determine the proteasomal susceptibility of the stable dimeric versus monomeric/dimerization intermediate forms of wtNSP3 by determining the half-lives of these forms of the protein in cells treated with or without proteasome inhibitor. We infected BSC-1 cells with vNSP3 to express wtNSP3 and added MG132 or its diluent DMSO 12 h prior to the addition of cycloheximide (CHX) to inhibit host cell translation at 14 hpi (time 0). Thereafter, a number of replicas were harvested and analyzed by WB-ND to determine the decay of wtNSP3 in the absence of protein synthesis. As shown in Fig 6A, the presence of MG132 stabilized wtNSP3 monomers/dimerization intermediates and stable dimers. As estimated from the data of three independent experiments (Fig 6B), in the absence of MG132, stable NSP3 dimers decayed sharply, with a half-life of 60 min, and monomers/dimerization intermediates decayed at a slower rate, with a half-life of 134 min. Surprisingly, stable dimers were more susceptible to the proteasome than monomers/dimerization intermediates, as determined by comparison of their half-lives in the presence or absence of the proteasome inhibitor. These data indicate that both stable dimeric and monomeric/dimerization intermediate forms of wtNSP3 are degraded via the proteasome, and stable dimeric NSP3 has a shorter half-life than monomers/dimerization intermediates. It is of note that even after 240 minutes in the presence of MG132 and CHX the amount of monomers/dimerization intermediates increased slightly and stable dimers increased to a higher extent, with a high standard deviation. If indeed the amount of NSP3 raises in the presence of CHX, it may be due to the high efficiency of translation of its IRES-containing mRNA, before the effect of CHX is fully stablished, combined with the extremely long half-lives of both forms of the protein in the presence of a proteasome inhibitor.

## Discussion

### Regulated expression of RVA NSP3 in the vaccinia virus VOTE system is an effective approach to assay its inhibitory effect on host cell translation

It is widely believed that RVA NSP3 inhibits host cell translation by disrupting the 5’ to 3’ mRNA interaction of capped and polyadenylated cellular mRNAs. This mechanism is now under scrutiny based on recent findings suggesting the opposite role of NSP3 as a surrogate of PABP that enhances polyadenylated mRNA translation by stabilizing eIF4E-eIF4G interaction [9, 12]. We hypothesized that the process of dimerization is a key to understand the mechanism by which NSP3 affects host cell translation. To begin to study the process of NSP3 dimerization, we performed mutagenesis of conserved amino acids in a segment of the coiled-coil region of this protein. We used the vaccinia virus VOTE system, a method we previously used to demonstrate that RRV NSP3 inhibits host cell translation. We confirmed that expression of wtNSP3 inhibits host cell translation (Fig 2), achieving a maximum 10-fold reduction that is below the 25-fold reduction obtained previously using hypertonic conditions during metabolic labelling [11]. In spite of the quantitative difference on host cell translation inhibition compared with prior studies, the VOTE system consistently achieved host cell translation inhibition similar to that obtained in RVA infected cells. Moreover, the VOTE system allows temporal regulation of expression [18], a feature that makes it suitable to mimic some of the conditions that occur in RVA infected cells. Notably, the conditions used in this study were chosen to maximize the inhibitory effect of RRV NSP3 on host cell translation, but complete shutoff of host cell translation did not occur.

### Point mutations in the coiled-coil of RRV NSP3 affect dimerization and stability to the proteasome

We observed in Fig 4 that four site-directed mutations reduced the level of NSP3 accumulation in comparison with wtNSP3 by affecting primarily the yield of stable dimers and to a lower extent the yield of monomers/dimerization intermediates (W170A, K171E, R173E and R187E:K191E). All mutations targeted a short 23 amino acids segment predicted to affect interactions between the two helices of a coiled-coil; hence, these mutations are likely affecting dimer formation. Based on these data, we conclude that four mutations affected primarily dimer formation and had a minor side effect on the yield of monomers/dimerization intermediates, possibly as a consequence of differential stability to the proteasome of the different forms of the protein.

This study confirms and expands prior studies indicating that degradation via the proteasome is the major determinant of NSP3 stability. We found that a proteasome inhibitor stabilized both stable NSP3 dimers and monomers/dimerization intermediates (Fig 6), thus demonstrating that stable NSP3 dimers and dimerization intermediates/monomers are targets of the proteasome. Moreover, the proteasome inhibitor enhanced the accumulation of high molecular weight forms of NSP3 migrating directly above the stable dimers (Fig 5), thus suggesting that the smears consists of polyubiquitinated dimers of this protein. A second band of approximately 30 kDa accumulated to a higher level in the presence of the proteasome inhibitor (Fig 5), however it is not clear how the proteasome inhibitor could enhance the yield of a putative degradation product of NSP3. Furthermore, our results indicate that stable NSP3 dimers have a shorter half-life than dimerization intermediates/monomers (Fig 6). The results of others indicates that *bona fide* NSP3 monomers are highly susceptible to proteasome degradation and are protected from such degradation as a consequence of binding to Hsp90, *i*. *e*. dimerization intermediates are more stable to the proteasome than *bona fide* monomers [17]. Taken together, these data suggest that NSP3 becomes a target of proteasome degradation immediately after its synthesis and remain as a target after reaching the dimerization intermediate and stable dimer forms, *i*. *e*. throughout dimerization. In this study, the ratio of *bona fide* monomers versus dimerization intermediates that blend together to form the corresponding band in WB-ND is not known, however the finding that such band has a relatively long half-life suggests that it is made mostly of dimerization intermediates, considering that this form of the protein is more stable to the proteasome than *bona fide* monomers [17]. Of note, one of the four mutations that affected dimer formation, R187E:K191E, included two mutations that have been tested individually on binding of NSP3 to the cellular protein RoXaN by co-immunoprecipitation; however, the dimerization and stability of these mutant proteins to the proteasome were not determined [14]. The potential effect of the coiled-coil mutations on the dimerization and stability of NSP3 merits caution in the interpretation of co-immunoprecipitation assays suggesting that these mutations affect the interaction of the mutant proteins with RoXaN.

In contrast with four mutations that affected NSP3 dimer formation, another mutation had the opposite effect (Y192A), it increased the level of total NSP3 accumulation by enhancing the yield of both stable dimers and monomers/dimerization intermediates (Figs 4 and 5). Considering that our results and those of others suggest that NSP3 becomes a target of proteasome degradation as soon as it is synthesized and remains as a proteasome target throughout dimerization, the observed effect of the mutation Y192 on the accumulation of all forms of the protein could be obtained if this mutation stabilizes *bona fide* monomers to the proteasome, thus increasing the accumulation of all forms of the protein.

Our mutagenesis approach targeted a segment of the coiled-coil region to affect NSP3 dimerization. The finding that four of the five mutations assayed affected the dimerization phenotype was unexpected considering the length of the coiled-coil region. Compensatory effects of other coiled-coil interactions are likely to render the mutations inconsequential. An alternative mechanism that would undermine stable dimer formation is a defect in the conformational rearrangement that Hsp90 triggers in its client proteins using energy from ATP to drive the process. Further studies are needed to assess whether NSP3 dimers are highly susceptible to single amino acid mutations in their lengthy coiled-coil interface or a conformational rearrangement during the course of dimerization is affected by such mutations.

### The level of stable dimeric RRV NSP3 does not correlate with its inhibitory function in host cell translation

**Fig 7:**
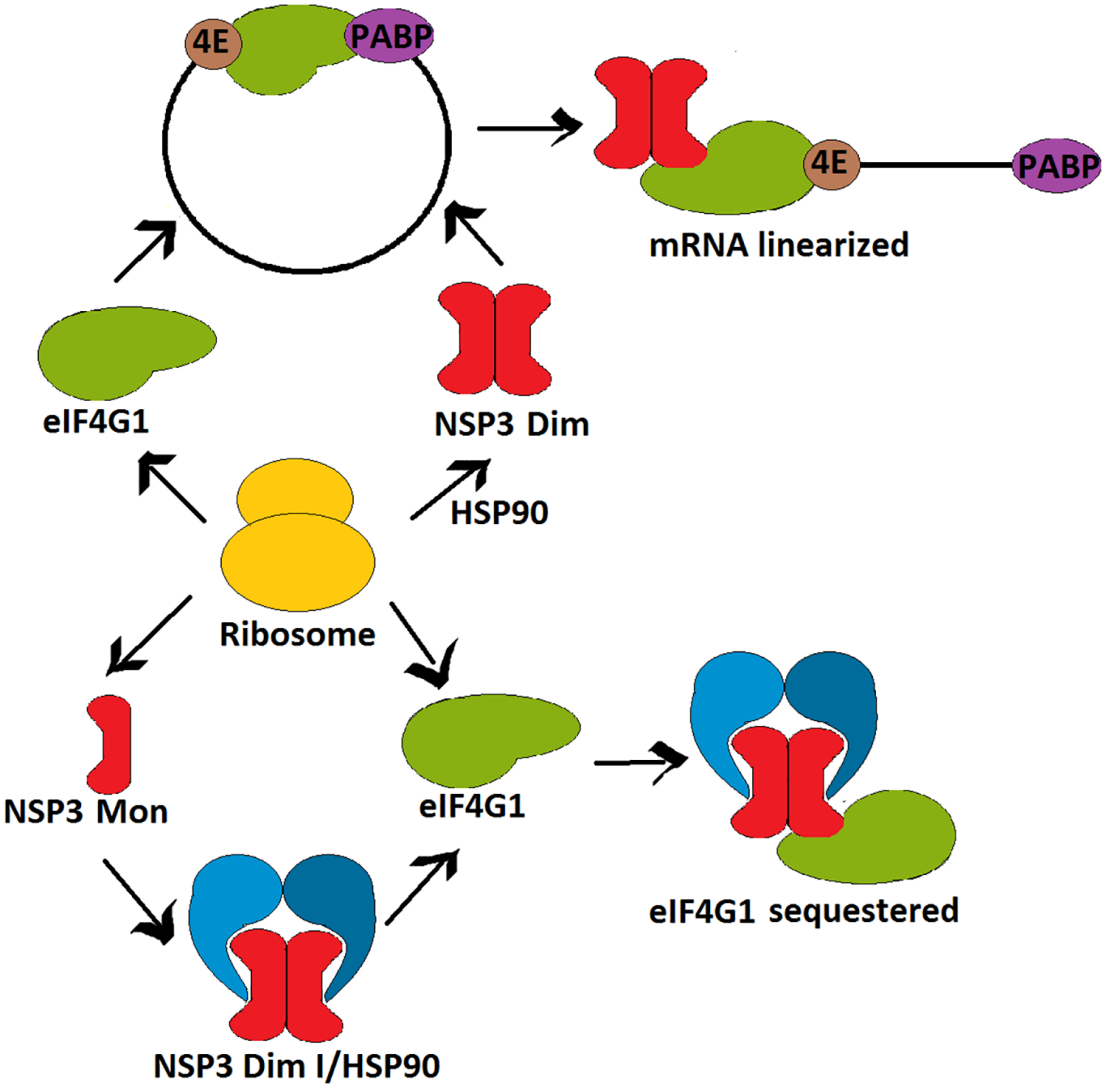
A model of RVA NSP3 translation inhibition prior to stable dimer formation. The current model (above) propose that mature NSP3 dimers engage eIF4F-mRNA-PABP complexes thus disrupting 5’ to 3’ mRNA interaction. Alternatively, NSP3 dimerization intermediates bound to HSP90 (below) would sequester eIF4G1.

We observed variable levels of accumulation of the stable dimeric forms of the five mutants of NSP3 compared with wtNSP3. Four mutants yielded lower levels of stable dimer accumulation, with the extreme case of mutation W170A, which exhibited a nine-fold difference with wtNSP3 and a sixteen-fold difference with NSP3_Y192A_ (Figs 4 and 5). It was surprising that the inhibitory function of NSP3 did not correlate with the level of accumulation of the stable dimeric form, as it was observed by comparison of the effect of mutations Y192A and W170A that inhibited host cell translation similarly in spite of the vast difference in their levels of accumulated stable dimeric protein. Because mutants with severe deficiency to form stable NSP3 dimers were still able to inhibit host cell translation significantly, we hypothesized that at least a fraction of the protein detected by WB-ND as monomers/dimerization intermediates is competent to inhibit host cell translation. In spite that we were unable to determine the ratio of *bona fide* monomers versus dimerization intermediates that blend together to form the corresponding band in WB-ND, the competence of three mutants with severely reduced levels of stable NSP3 dimers (W170A, R173E, and R187E:K191E) to inhibit host cell translation, suggest that a significant fraction of the dimerization intermediates/monomers detected by WB-ND are functional dimerization intermediates. This hypothesis is consistent with observed long half-life of this form of the protein, as opposed to the short-lived *bona fide* monomers [17]. Based on these findings, we hypothesize that NSP3 inhibits host cell translation by sequestering eIF4G1 along with such dimerization intermediates rather than by disrupting the 5’ to 3’ mRNA interaction (Fig 7).

In this study, we demonstrate that RVA NSP3 acquires its function prior to stable dimer formation and remain as a proteasome target throughout dimerization. Further studies of the macromolecular complexes involved in NSP3 dimerization and in targeting for proteasome degradation are needed to better understand these processes.

## Supporting information

S1 Fig Effect on host cell protein synthesis of wild-type RRV NSP3 and five site-directed NSP3 mutants expressed with recombinant vaccinia viruses using different doses of the inducer IPTG. BSC-1 cells were infected with the parental virus vT7lacOI, or with viruses for the expression of wild type NSP3 or its mutants with a MOI of five. At two hpi, different doses of IPTG were added. At 17 hpi the infected cells were pulse-labeled with [^35^S]-methionine plus [^35^S]-cysteine for one hour and harvested. The cells were then analyzed by SDS-PAGE and autoradiography (A and B). The molecular weights of four predominant vaccinia virus proteins detected in cells infected with vT7lacOI are indicated to the left. The solid triangles indicate the position of the NSP3 bands (34 kDa). Based on densitometry analysis of three independent experiments, the graph bars (C) indicate the percentage of protein synthesis in cells expressing NSP3 or its mutants compared with control cells that do not express NSP3 (vT7). Bars indicate standard deviation.
(TIF)

S2 Fig Effect on host cell protein synthesis of different times of induction of RRV NSP3 expressed with a recombinant vaccinia virus. BSC-1 cells were mock-infected, infected with the parental virus vT7lacOI, or with vNSP3 for the expression of wild type NSP3 with a MOI of five. At two, 6 or 10 hpi the inducer IPTG was added (0.4 mM). At 17 hpi the infected cells were pulse-labeled with [^35^S]-methionine plus [^35^S]-cysteine for one hour and harvested. The cells were then analyzed by SDS-PAGE and autoradiography (A). Mock-infected cells are indicated in the first lane (M). The molecular weights of four predominant vaccinia virus proteins detected in cells infected with vT7lacOI are indicated to the left. The solid triangle indicates the position of the NSP3 band (34 kDa). Based on densitometry analysis of A, the graph bars (B) indicate the percentage of protein synthesis in cells expressing NSP3 compared with control cells that do not express NSP3 (vT7).
(TIF)
