# Supplementary figures and images for: Species A rotavirus NSP3 acquires its translation inhibitory function prior to stable dimer formation
Source: PLoS One. 2017 Jul 24;12(7):e0181871. doi: 10.1371/journal.pone.0181871 (PMC5524322; doi:10.1371/journal.pone.0181871)

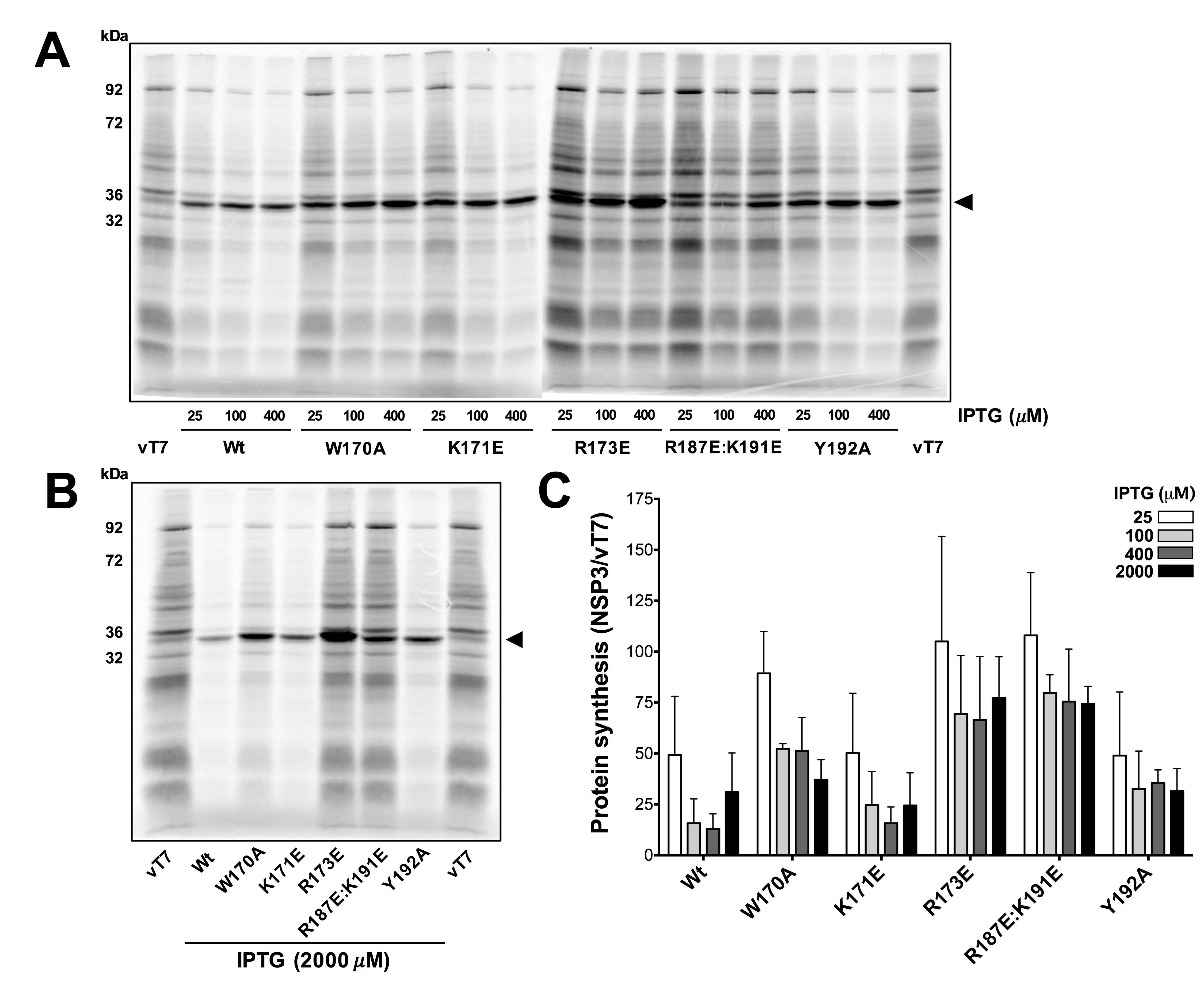

Supplement: S1 Fig — BSC-1 cells were infected with the parental virus vT7lacOI, or with viruses for the expression of wild type NSP3 or its mutants with a MOI of five. At two hpi, different doses of IPTG were added. At 17 hpi the infected cells were pulse-labeled with [35S]-methionine plus [35S]-cysteine for one hour and harvested. The cells were then analyzed by SDS-PAGE and autoradiography (A and B). The molecular weights of four predominant vaccinia virus proteins detected in cells infected with vT7lacOI are indicated to the left. The solid triangles indicate the position of the NSP3 bands (34 kDa). Based on densitometry analysis of three independent experiments, the graph bars (C) indicate the percentage of protein synthesis in cells expressing NSP3 or its mutants compared with control cells that do not express NSP3 (vT7). Bars indicate standard deviation. (TIF) [file pone.0181871.s001.tif]

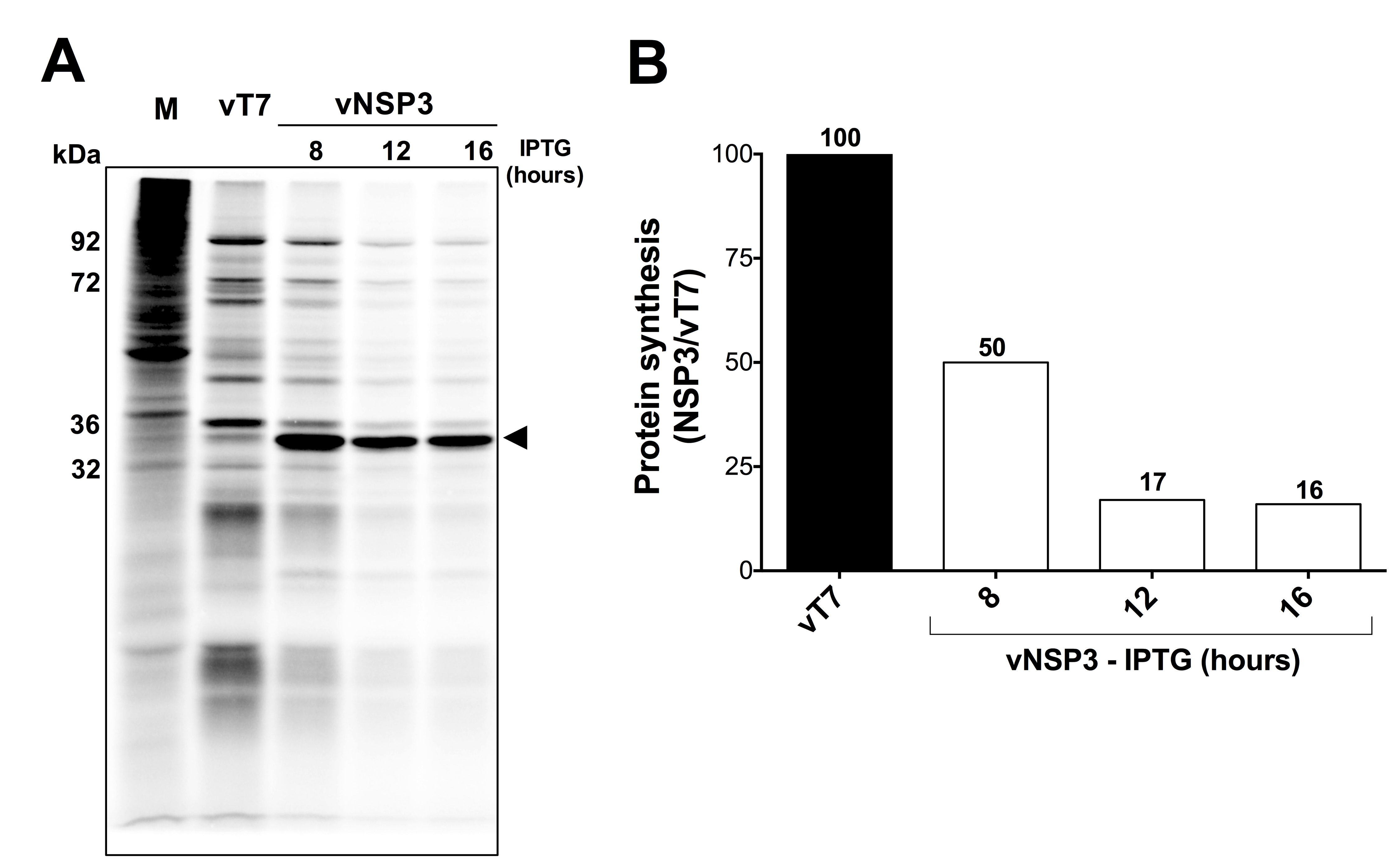

Supplement: S2 Fig — BSC-1 cells were mock-infected, infected with the parental virus vT7lacOI, or with vNSP3 for the expression of wild type NSP3 with a MOI of five. At two, 6 or 10 hpi the inducer IPTG was added (0.4 mM). At 17 hpi the infected cells were pulse-labeled with [35S]-methionine plus [35S]-cysteine for one hour and harvested. The cells were then analyzed by SDS-PAGE and autoradiography (A). Mock-infected cells are indicated in the first lane (M). The molecular weights of four predominant vaccinia virus proteins detected in cells infected with vT7lacOI are indicated to the left. The solid triangle indicates the position of the NSP3 band (34 kDa). Based on densitometry analysis of A, the graph bars (B) indicate the percentage of protein synthesis in cells expressing NSP3 compared with control cells that do not express NSP3 (vT7). (TIF) [file pone.0181871.s002.tif]
